# Modulation of spinal cord synaptic activity by tumor necrosis factor α in a model of peripheral neuropathy

**DOI:** 10.1186/1742-2094-8-177

**Published:** 2011-12-21

**Authors:** Diana Spicarova, Vladimir Nerandzic, Jiri Palecek

**Affiliations:** 1 Department of Functional Morphology, Institute of Physiology, Academy of Sciences of the Czech Republic, Prague, Czech Republic

**Keywords:** axotomy, sciatic nerve, dorsal horn, synaptic transmission, TRPV1, sodium channels

## Abstract

**Background:**

The cytokine tumor necrosis factor α (TNFα) is an established pain modulator in both the peripheral and central nervous systems. Modulation of nociceptive synaptic transmission in the spinal cord dorsal horn (DH) is thought to be involved in the development and maintenance of several pathological pain states. Increased levels of TNFα and its receptors (TNFR) in dorsal root ganglion (DRG) cells and in the spinal cord DH have been shown to play an essential role in neuropathic pain processing. In the present experiments the effect of TNFα incubation on modulation of primary afferent synaptic activity was investigated in a model of peripheral neuropathy.

**Methods:**

Spontaneous and miniature excitatory postsynaptic currents (sEPSC and mEPSCs) were recorded in superficial DH neurons in acute spinal cord slices prepared from animals 5 days after sciatic nerve transection and in controls.

**Results:**

In slices after axotomy the sEPSC frequency was 2.8 ± 0.8 Hz, while neurons recorded from slices after TNFα incubation had significantly higher sEPSC frequency (7.9 ± 2.2 Hz). The effect of TNFα treatment was smaller in the slices from the control animals, where sEPSC frequency was 1.2 ± 0.2 Hz in slices without and 2.0 ± 0.5 Hz with TNFα incubation. Tetrodotoxin (TTX) application in slices from axotomized animals and after TNFα incubation decreased the mEPSC frequency to only 37.4 ± 6.9% of the sEPSC frequency. This decrease was significantly higher than in the slices without the TNFα treatment (64.4 ± 6.4%). TTX application in the control slices reduced the sEPSC frequency to about 80% in both TNFα untreated and treated slices. Application of low concentration TRPV1 receptors endogenous agonist *N*-oleoyldopamine (OLDA, 0.2 μM) in slices after axotomy induced a significant increase in mEPSC frequency (175.9 ± 17.3%), similar to the group with TNFα pretreatment (158.1 ± 19.5%).

**Conclusions:**

Our results indicate that TNFα may enhance spontaneous transmitter release from primary afferent fibres in the spinal cord DH by modulation of TTX-sensitive sodium channels following sciatic nerve transection. This nerve injury also leads to enhanced sensitivity of presynaptic TRPV1 receptors to endogenous agonist. Modulation of presynaptic receptor activity on primary sensory terminals by TNFα may play an important role in neuropathic pain development.

## Background

It is now well established that neuroinflammation can facilitate or directly produce pain due to increased release of different cytokines which in turn recruit immune cells and activate glial cells [1,2], especially under neuropathic conditions [3,4]. The cytokine tumor necrosis factor α (TNFα) is now recognized as a pain modulator participating in both the peripheral and central processes leading to neuropathic pain following peripheral nerve injury [5]. Several studies demonstrated increased TNFα levels in DRG [6-8] and spinal cord [9-11] in different models of peripheral neuropathy. The main sources of cytokines in the spinal cord DH are activated glial cells [1,10-12]. Recently, it was shown that acute application or incubation of spinal cord slices with TNFα modulates excitatory [13-16] and inhibitory [17,18] synaptic transmission in the spinal cord DH. Furthermore, intrathecal application of exogenous TNFα induced mechanical allodynia and thermal hyperalgesia in rats and mice [13-15]. Pain hypersensitivity associated with peripheral neuropathy was attenuated by the TNFα antagonist etanercept [19,20].

The effect of TNFα is mediated by two receptors, the TNFR1 (p55) and the TNFR2 (p75). Both receptors were detected in DRG and spinal cord neurons [21,22]. Up-regulation of TNFR1/2 receptors in DRG neurons [23-25] and TNFR1 in the spinal cord DH [11,26] was demonstrated in different models of peripheral neuropathy. Presumably, TNFR1 and TNFR2 differentially regulate nociceptive signaling. A crucial role of peripheral TNFR1 receptors activation was demonstrated in the CCI model of neuropathy [27]. In addition, using TNFR1 or TNFR2 knockout mice it was shown that development of thermal hyperalgesia after CCI depended upon the TNFR1 gene, whereas mechanical allodynia was present in both TNFR1 or TNFR2 knockout mice [28]. The importance of TNFR2 receptors in the excitation of primary sensory neurons after spinal nerve ligation (SNL) was demonstrated using proteins that selectively activate TNFR1 or TNFR2 receptors [29]. The same study indicated a decrease in mechanical and thermal withdrawal thresholds induced by intrathecal injection of a selective TNFR1 but not TNFR2 agonist in control rats, whereas coinjection of both selective agonists induced robust pain hypersensitivity [29]. By using TNFR1 and TNFR2 knockout mice it was shown that thermal hyperalgesia induced by intrathecal injection of TNFα could be mediated by both TNFR1 or TNFR2 receptors, but it was completely abolished in TNFR1/2 double knockout mice [15]. TNFα application evoked an increase in the spontaneous EPSC frequency in the superficial DH neurons, which was eliminated in the TNFR1 knockout mice and reduced in the TNFR2 -/- mice [15].

Voltage activated sodium channels (Nav), especially the tetrodotoxin-sensitive (TTX-S) Nav 1.3 and tetrodotoxin-resistant (TTX-R) Nav 1.8 channels were implicated in neuropathic pain states [30]. It was demonstrated that following sciatic nerve axotomy, expression of Nav 1.3 channel mRNA was up-regulated, while Nav 1.8 mRNA was down-regulated in small DRG neurons [31,32]. This corresponds well to the observed four times faster recovery from inactivation of TTX-S sodium currents in axotomized than control small DRG neurons and down-regulation of TTX-R currents [33]. Nav 1.3 channels are present in embryonic, but not in adult DRG neurons and are re-expressed under pathological condition [31]. In spinal nerve ligation (SNL) neuropathy, mechanical allodynia and thermal hyperalgesia were attenuated with reduction of TTX-R current in DRG neurons by specific knockdown of Nav 1.8 with antisense oligodeoxynucleotides [34]. Antisense oligodeoxynucleotides targeting Nav 1.8 also attenuated hypersensitivity in the chronic constriction injury (CCI) model [35]. In injured DRG neurons Nav 1.8 protein expression decreased [36], but there was an increase in Nav 1.8 immunoreactivity along the sciatic nerve following SNL [37]. It was proposed that Nav 1.8 channels in uninjured DRG neurons contribute to the hyperexcitability of these neurons, which may be critical for the development of neuropathic pain [37]. These studies indicate that the expression of Nav channels and their function in primary afferent neurons could be differentially regulated in injured and in uninjured neurons, suggesting that up-regulation of TTX-S Nav 1.3 channels is crucial in injured neurons whereas increases of TTX-R Nav 1.8 channels are more important in uninjured DRG neurons during neuropathy. Interestingly, TNFα may affect expression of both channels as peri-sciatic administration of TNFα up-regulated Nav 1.3 and 1.8 in DRG neurons [38].

Transient receptor potential vanilloid 1 receptors (TRPV1) are well recognized as molecular integrators of nociceptive stimuli in the periphery. Recently, the presynaptic TRPV1 receptors on the central branches of primary afferent neurons in the spinal cord were shown to have important roles in nociceptive synaptic signaling especially under pathological conditions [39-41]. Coexpression of TNFR1 and TRPV1 receptor mRNA [42] and colocalization of TNFR1 and TRPV1 immunoreactivity [43] was reported in subsets of DRG neurons. TNFα enhanced the sensitivity of cultured DRG neurons to capsaicin application [44] and induced increased expression of TRPV1 receptors on DRG [43] and trigeminal ganglion neurons [45]. This increased expression of TRPV1 receptors was TNFR1 dependent in naïve mice [43] in contrast to tumor-bearing mice, where up-regulation of TRPV1 receptors was dependent on TNFR2 [46]. In our previous experiments we have demonstrated increased sensitivity of presynaptic TRPV1 receptors to the endogenous vanilloid agonist *N*-oleoyldopamine (OLDA) after TNFα treatment in spinal cord slices from control animals [16]. The absence of DRG in our preparation indicated that this effect was due to phosphorylation of native TRPV1 receptors as opposed to their increased expression [47]. It was shown that capsaicin-evoked current was robustly potentiated via activation of PKC or p38/MAP kinase after TNFα application in cultured DRG neurons [46].

In the present study, we have examined the modulation of synaptic transmission by TNFα in the superficial spinal cord DH after sciatic nerve transection and in control animals. The effect of acute slice incubation with TNFα on the spontaneous and miniature EPSCs and on TRPV1 receptor activation by the endogenous agonist OLDA was investigated.

## Methods

All experiments were approved by the local Institutional Animal Care and Use Committee and were consistent with the guidelines of the International Association for the Study of Pain, the National Institutes of Health Guide for the Care and Use of Laboratory Animals and the European Communities Council Directive of 24 November 1986 (86/609/EEC).

### Sciatic nerve transection

Male Wistar rats on postnatal day P18 to P22 were anesthetized with ether. For axotomy both sciatic nerves were exposed at midthigh level and transected using sharp scissors. The wound was closed and animals were left to recover in their home cages.

### Spinal cord slices preparation

Acute spinal cord slices were prepared from male Wistar rats P20 - P27, as was previously described [39]. After anesthesia with ketamine (150 mg/kg, i.p.) and xylazine (16 mg/kg, i.p.), the lumbar spinal cord was removed and immersed in oxygenated ice-cold dissection solution containing (in mM): 95 NaCl, 1.8 KCl, 7 MgSO_4_, 0.5 CaCl_2_, 1.2 KH_2_PO_4_, 26 NaHCO_3_, 25 D-glucose, 50 sucrose. The spinal cord was fixed to a vibratome stage (Leica, VT 1000 S, Germany) in a groove between two agar blocks using cyanoacrylate glue. Acute transverse slices 300 μm thick were cut from lumbar segments L_3_-L_5_, incubated in the dissection solution for 30 min at 33°C and then stored in a recording solution at room temperature and allowed to recover for 1 h before the electrophysiological experiments. Recording solution contained (in mM): 127 NaCl, 1.8 KCl, 1.2 KH_2_PO_4_, 2.4 CaCl_2_, 1.3 MgSO_4_, 26 NaHCO_3_, 25 D-glucose. In some experiments the slices were incubated for at least 2 h with TNFα (60 nM added in the bath). Electrophysiological measurements were made from slices transferred into a recording chamber that was perfused continuously with recording solution at a rate ~2 ml/min. All extracellular solutions were saturated with carbogen (95% O_2_, 5% CO_2_) during the whole process.

### Electrophysiological recordings

Patch-clamp recordings were made from individual DH neurons visualized using a differential interference contrast (DIC) microscope (Leica, DM LFSA, Germany) equipped with an infrared-sensitive camera (IR camera Hitachi KP-200 P, Japan) with a standard TV/video monitor (Hitachi VM-172, Japan). Patch pipettes were pulled from borosilicate glass tubing (Rückl Glass, Otvovice, Czech Republic) with resistances of 3.5 - 6.0 MΩ when filled with intracellular solution. The intracellular pipette solution contained (in mM): 125 gluconic acid lactone, 15 CsCl, 10 EGTA, 10 HEPES, 1 CaCl_2_, 2 Na_2_ATP, 0.5 NaGTP and was adjusted to pH 7.2 with CsOH. Voltage-clamp recordings in the whole-cell configuration were performed with an Axopatch 200 B amplifier and 1440 A digitizer (Molecular Devices, USA) at room temperature (~23°C). Whole-cell responses were low-pass filtered at 2 kHz and digitally sampled at 10 kHz. The series resistance of neurons was routinely compensated by 80% and was monitored during the whole experiment. AMPA receptor-mediated spontaneous or miniature EPSCs were recorded from superficial DH neurons in laminae I and II, clamped at -70 mV in the presence of 10 μM bicuculline and 5 μM strychnine. Miniature EPSCs were distinguished by addition of 0.5 μM tetrodotoxin (TTX) to the recording solution. Lidocaine (1 mM) was added in other experiments to block TTX-R sodium channels. The software package pCLAMP version 10 (Molecular Devices, USA) was used for data acquisition and subsequent off-line analysis. Neurons with capsaicin-sensitive primary afferent input were identified by an increase of EPSC frequency (> 20%) following capsaicin (0.2 μM) administration at the end of the experimental protocol.

### Drug treatment

All drugs used in this study were of analytical grade and purchased from Sigma-Aldrich (Prague, Czech Republic) or Tocris Bioscience (Bristol, UK). TNFα was dissolved in 0.1% BSA; capsaicin and OLDA were dissolved in dimethylsulfoxide (DMSO), which had a concentration < 0.1% in the final solution.

### Data analysis

Data segments of 2 min duration were analyzed for each experimental condition. Only EPSCs with an amplitude of 5 pA or greater (which corresponded to at least twice the recording noise level) were included in the frequency analysis. In the case of amplitude analysis, the same events and data segments were used. Data are expressed as means ± standard error of the mean (SEM). Some data were normalized as a percentage of the control values (100%). Paired t-test, one-way ANOVA or one-way ANOVA repeated measures followed by post hoc test (Bonferroni) were used for statistical comparisons and P < 0.05 was considered to be statistically significant.

## Results

**Figure 1:**
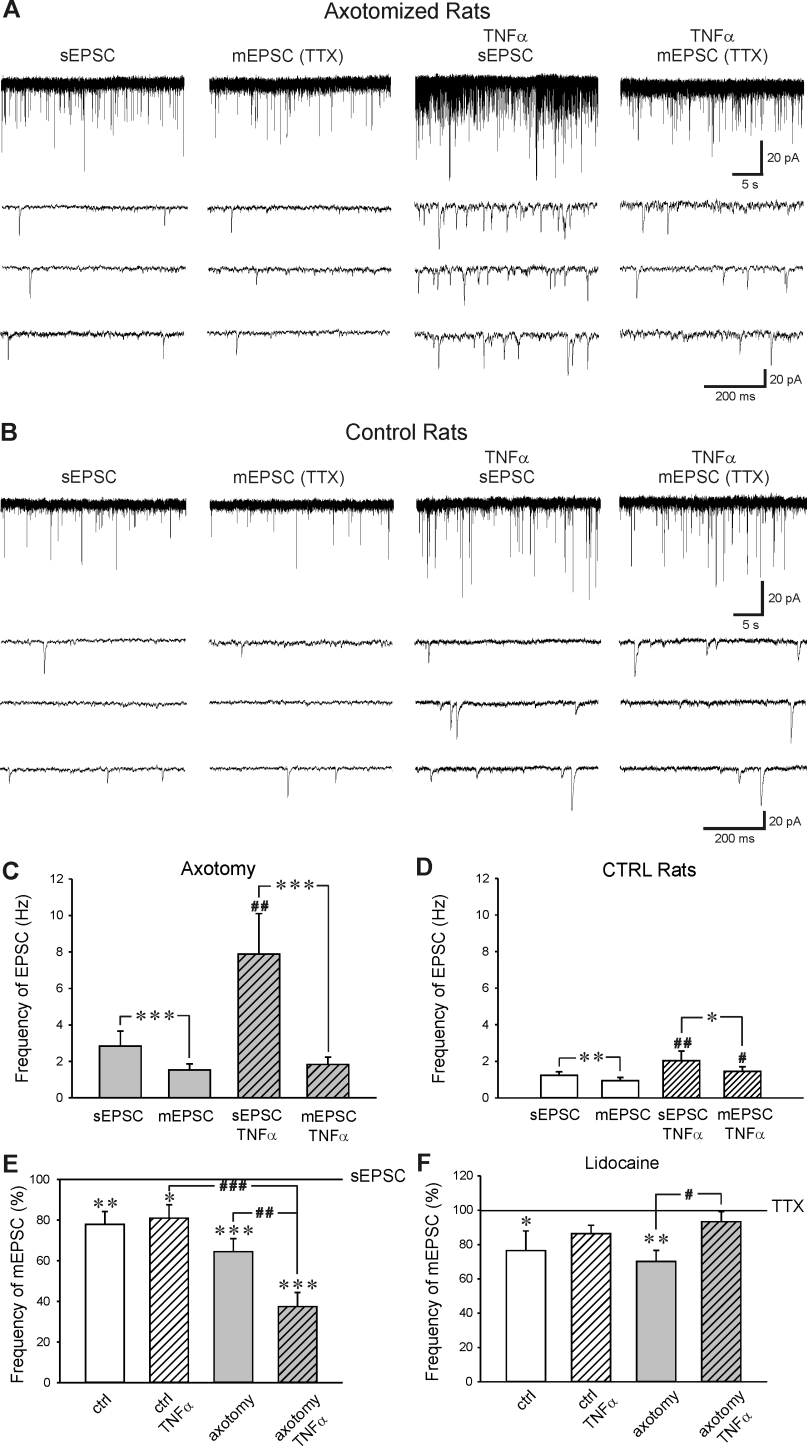
**Example of sEPSC and mEPSC activity recorded in neurons after axotomy **(A) **and in control animals **(B) **without and after TNFα treatment**. **C**) TNFα (60 nM) pretreatment robustly increased the frequency of sEPSC in slices after sciatic nerve section (n = 12, ^##^P < 0.01) compared to non-pretreated slices (n = 18). Mean mEPSC frequency was similar after acute tetrodotoxin (TTX, 0.5 μM) application in both groups, with and without TNFα treatment and significantly reduced compared to the sEPSC frequency (***P < 0.001). **D) **Mean sEPSC (n = 9, ^##^P < 0.01) and mEPSC (^#^P < 0.05) frequency was significantly higher in control animals after TNFα treatment. TTX application reduced sEPSC frequency significantly in both TNFα treated (*P < 0.05) and non-treated slices (**P < 0.01). **E) **TTX application dramatically decreased sEPSC frequency in spinal cord slices after axotomy pretreated with TNFα (n = 12). This TNFα induced TTX dependent decrease of sEPSC frequency was not present in control slices (n = 9). TTX induced decrease of sEPSC frequency in slices after axotomy without TNFα treatment (n = 18) was not statistically different from the results in the control group (n = 20). *: comparison of mEPSC versus sEPSC; #: comparison of TNFα treated sEPSC and mEPSC versus non-treated sEPSC and mEPSC, respectively. **F**) Lidocaine application reduced mEPSC frequency present during TTX application in neurons from control (n = 6) and axotomized (n = 7) slices. Effect of lidocaine application was not significant after TNFα treatment in the neurons recorded in the control (n = 8) and axotomized (n = 8) slices.

Five days after the sciatic nerve transection spontaneous and miniature AMPA EPSCs were recorded in spinal cord slices without and after incubation with TNFα (60 nM). The absolute sEPSC frequency in the DH neurons after axotomy was 2.83 ± 0.83 Hz and decreased to 1.52 ± 0.34 Hz (n = 18, P < 0.001) after TTX application (Figure 1A, C). In slices from control animals TTX application reduced sEPSC frequency from 1.23 ± 0.20 Hz to 0.94 ± 0.17 Hz (n = 20, P < 0.01, Figure 1B, D). Spontaneous and mEPSC frequency in DH neurons in slices after axotomy was higher than in control animals, but this difference was not statistically significant. In slices after axotomy incubated with TNFα, the absolute sEPSC frequency was 7.89 ± 2.21 Hz and decreased to 1.83 ± 0.40 Hz (n = 12, P < 0.001) after TTX application (Figure 1A, C). Spontaneous EPSC frequency in TNFα incubated slices in control animals decreased due to TTX application from 2.03 ± 0.53 Hz to 1.45 ± 0.25 Hz (n = 9, P < 0.05, Figure 1B, D). There was evident TNFα mediated increase of the sEPSC frequency in slices after sciatic nerve transection when compared to control slices (P < 0.05), while the low difference between mEPSC in these two groups was not significant (Figure 1C, D). These results indicate that TNFα increases sEPSC frequency in axotomized DH neurons via enhanced activity at TTX-S Nav channels. This is even more evident after standardization of the results. Under this evaluation, tetrodotoxin application reduced the frequency of spontaneous EPSC to 64.4 ± 6.4% (n = 18, P < 0.001) in neurons after axotomy without TNFα treatment, but these were decreased to only 37.4 ± 6.9% (n = 12, P < 0.001) with TTX application in spinal cord slices pretreated with TNFα (Figure 1E). This robust TTX induced decrease of EPSC frequency in TNFα pretreated slices was statistically different from the TTX effect in non-pretreated slices in the axotomy group (P < 0.01) and from the TTX effect in control animals (P < 0.001). There was no difference between the TTX induced reductions of EPSC frequency in neurons incubated with TNFα (80.9 ± 6.6, n = 9, P < 0.05) and non-pretreated slices (77.8 ± 6.4, n = 20, P < 0.01) in control animals.

**Figure 2:**
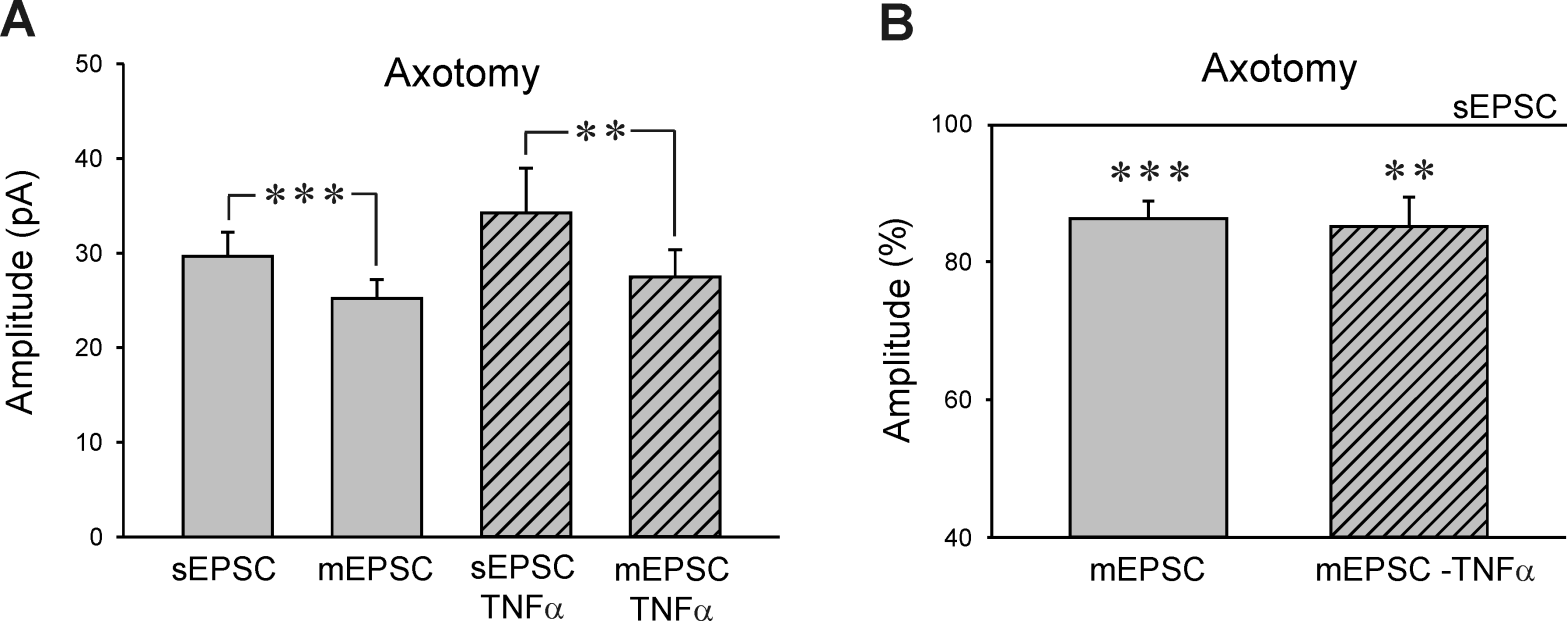
**A) The mean amplitude of sEPSC and mEPSC was not different in the axotomy groups of neurons with and without TNFα treatment**. Tetrodotoxin application (0.5 μM) decreased sEPSC amplitude in both TNFα treated (n = 12, **P < 0.01) and non-treated (n = 18, ***P < 0.001) superficial dorsal horn neurons after axotomy. **B) **The reduction of sEPSC amplitude after TTX application was similar in both TNFα treated (**P < 0.01) and non-treated (**P < 0.001) neurons.

The mean amplitude of the sEPSC was 29.7 ± 2.5 pA in the neurons after axotomy and decreased to 25.2 ± 2.0 pA (mEPSC) after TTX application (n = 18, P < 0.001, Figure 2A). In the group of TNFα pretreated neurons after axotomy the results were similar, with mean sEPSC amplitude of 35.2 ± 4.7 pA and mEPSC amplitude 28.6 ± 3.0 pA (n = 12, P < 0.01). In control animals, the mean sEPSC amplitude (29.7 ± 2.2 pA) decreased after TTX application to 25.9 ± 1.4 pA (n = 20, P < 0.05). Control TNFα pretreated neurons had sEPSC amplitude of 22.6 ± 1.9 pA and mEPSC of 21.1 ± 1.8 pA (n = 9). Neither sEPSC or mEPSC amplitudes were statistically different between the DH neurons in TNFα pretreated slices and non-treated slices from both injured and control animals. The standardized mean sEPSC amplitudes (100%) were higher than the mEPSC amplitudes in both axotomized and control slices, with and without TNFα treatment (Figure 2B). There was no difference in the TTX induced reduction of the mean sEPSC amplitude between the TNFα treated (85.1 ± 4.2%, n = 12, P < 0.01) and non-treated group (86.2 ± 2.6%, n = 18, P < 0.001) after axotomy (Figure 2B). In slices from the control rats, the TTX induced reduction of the mean sEPSC amplitude was only small, 94.7 ± 4.2% (n = 9) in the TNFα pretreated group and 91.1 ± 3.3% (n = 20, P < 0.05) in the slices without TNFα treatment.

To assess the role of TTX-R Nav channels, lidocaine (1 mM) was used together with TTX in some experiments. In slices from axotomized animals the mEPSC frequency decreased after lidocaine application to 70.1 ± 6.6% (n = 7, P < 0.01), when mEPSC frequency during the TTX application was considered 100% (Figure 1F). In axotomized slices with TNFα treatment, lidocaine application did not induce significant change in mEPSC frequency (93.3 ± 6.0%, n = 8) from the TTX level, but was significantly different from the TNFα untreated slices (P < 0.05). In DH neurons from control animals the mEPSC frequency decreased after lidocaine application to 76.5 ± 11.4% (n = 6, P < 0.05). The decrease of mEPSC frequency after lidocaine application in TNFα treated control slices (86.3 ± 4.9%, n = 8) was not statistically different from the frequency during the TTX application or from recordings in the control slices without TNFα treatment.

The mean amplitude of the mEPSCs recorded in the presence of lidocaine was not different from mEPSC amplitude recorded in the presence of TTX only (axotomy: 104.8 ± 3.5%, axotomy with TNFα: 103.1 ± 7.3%, CTRL: 97.7 ± 2.9%, CTRL with TNFα: 91.3 ± 3.3%).

**Figure 3:**
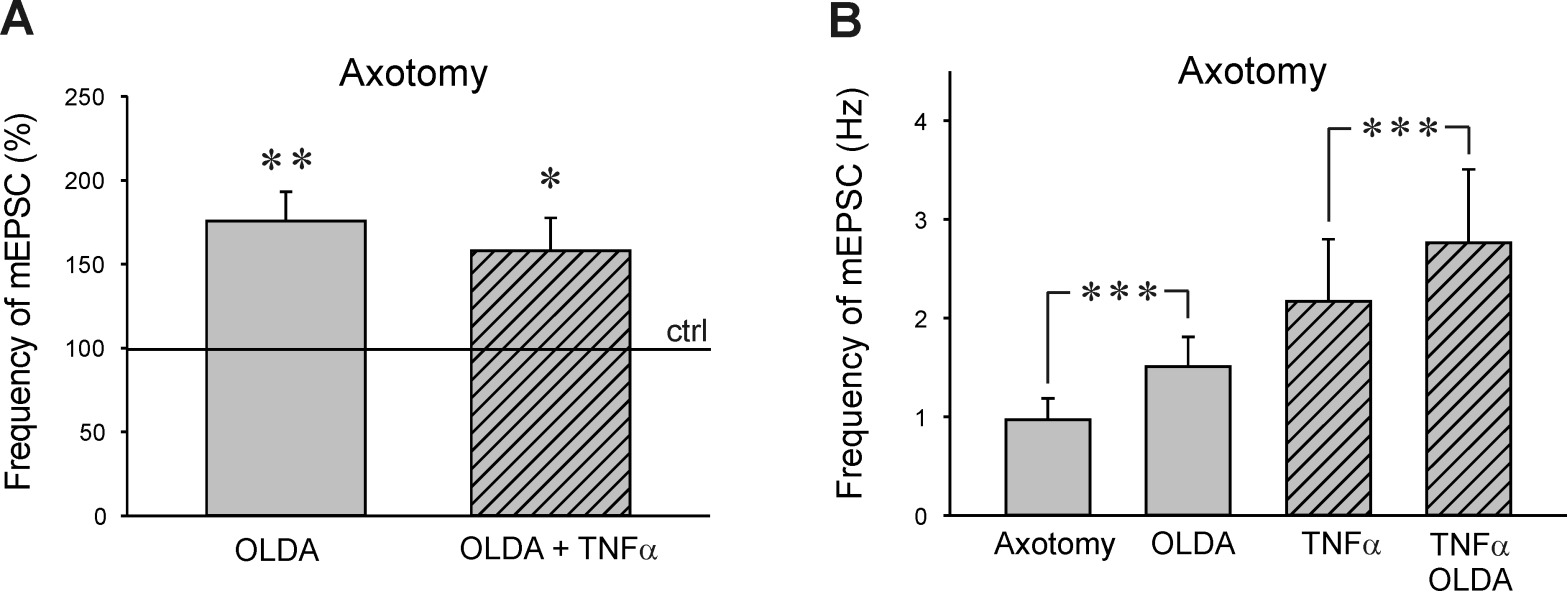
A) Neurons recorded in slices after axotomy showed increased mEPSC frequency after endogenous TRPV1 agonist *N*-oleoyldopamine application (OLDA, 0.2 μM, n = 13, **P < 0.01). The OLDA induced mEPSC frequency increase was similar in TNFα treated slices (n = 14, **P < 0.05). **B) **The absolute mEPSC frequency recorded in the neurons after TNFα treatment was higher compared to the non-treated group, but this difference did not reach statistical significance.

Next, TNFα modulation of spinal TRPV1 receptors activation by endogenous agonist OLDA after sciatic nerve transection was investigated. We have previously demonstrated that application of a low concentration OLDA (0.2 μM) does not evoke any changes in the mEPSC frequency in control slices [16,39]. This was also confirmed in the present experiments, where application of 0.2 μM OLDA solution did not change the mEPSC frequency (94.8 ± 5.0%, n = 6) in slices from control animals. However, the application of low concentration OLDA solution (0.2 μM) increased the mEPSC frequency to 175.9 ± 17.3% (n = 13, P < 0.01) when compared to the control values before the OLDA application, in acute spinal cord slices prepared from animals 5 days after the sciatic nerve transection (Figure 3A). Final capsaicin application (0.2 μM) increased the mEPSC frequency substantially (699.2 ± 426.9%), in this group of DH neurons. OLDA application in the neurons recorded in slices after axotomy and with the TNFα pretreatment increased the mEPSC frequency 158.1 ± 19.5% (n = 14, P < 0.05) and the capsaicin application increased mEPSC frequency to 860.2 ± 343.2%. OLDA induced increase of the mEPSC frequency was not statistically different between the TNFα pretreated and non-treated slices from the axotomized animals. The mEPSC frequency (in Hz) recorded in the TNFα incubated slices prepared from the animals after axotomy was higher (mEPSC: 2.17 ± 0.63 Hz, OLDA: 2.76 ± 0.75 Hz, n = 14) when compared to the mEPSC frequency recorded in slices without TNFα treatment (mEPSC: 0.97 ± 0.22 Hz, OLDA: 1.51 ± 0.30 Hz, n = 13, Figure 3B), but this difference was not statistically significant. All of the tested neurons responded to capsaicin application. OLDA application did not change the mean mEPSC amplitude in the recorded superficial DH neurons without (mEPSC: 23.7 ± 2.5 pA, OLDA: 23.6 ± 2.6 pA, n = 13) and with TNFα pretreatment (mEPSC: 28.0 ± 2.9 pA, OLDA: 25.9 ± 2.7 pA, n = 14) following peripheral nerve injury, similar to our results demonstrated in control animals [39].

## Discussion

There is now mounting evidence of TNFα importance in the processing of nociceptive information at the spinal cord level following peripheral nerve injury. In our study we have examined the possible role of TNFα in modulation of synaptic transmission at superficial DH neurons in a model of peripheral neuropathy. Our results showed increased TNFα mediated regulation of presynaptic TTX-S sodium channels activity, 5 days after sciatic nerve axotomy. The nerve injury also increased sensitivity of presynaptic TRPV1 receptors to endogenous agonist OLDA. These changes in function of presynaptic primary afferent ending in our experiments were most likely related to nociception *in vivo*, as the neurons recorded were in the superficial DH laminae and most of them received capsaicin sensitive input.

TNFα in our experiments induced robust increases of sEPSC frequency in the DH neurons recorded after the sciatic nerve transection, while in control animals the effect of TNFα incubation was only moderate. This TNFα induced sEPSC frequency increase after nerve injury was most likely mediated by increased expression of TNFR1 receptors and activation of TTX-S Nav channels, as TTX application reduced the sEPSC frequency to only 37% of the original level. The cytokines were shown to have significant impact on sodium channels activity *in vitro *and in different models of neuropathic pain. In cultured DRG neurons TNFα enhanced TTX-R sodium currents via activation of TNFR1 receptors and p38 MAPK [48]. Model of neuropathy induced by L5 ventral root transection (L5-VRT) accompanied with mechanical allodynia and thermal hyperalgesia increased immunoreactivity for TNFα and TNFR1 receptors in the ipsilateral DRG and bilaterally in the spinal cord DH [26]. Inhibition of TNFα synthesis in this model, prevented p38 MAPK activation in DRG neurons and spinal cord microglia, which was necessary for the initiation and maintenance of neuropathic pain [49]. The L5-VRT also increased Nav 1.3 and Nav 1.8 mRNA, protein level and current densities of TTX-S and TTX-R sodium channels in DRG neurons [38]. Interestingly, both Nav 1.3 and Nav 1.8 up-regulation was mediated by cytokine TNFα, as was shown by inhibition of TNFα synthesis [26,38]. The increase of sodium currents in DRG neurons evoked by L5-VRT was not present in TNRF1 knockout mice [50]. Our results in the peripheral nerve axotomy model suggest increased glutamate release from the presynaptic primary afferent endings in the spinal cord due to TTX-S Nav channels activity. The DRG cell bodies were absent during the incubation of spinal cord slices with TNFα. Therefore the increased spontaneous activity was most likely mediated through modulation of TTX-S Nav channels function, such as their phosphorylation/dephosphorylation [51], trafficking from the cytoplasm to the presynaptic membrane [52] or possibly also by their local synthesis in the presynaptic ending. Recently ERK1/2 mitogen-activated protein kinase phosphorylation of TTX-S Nav 1.7 channels was shown to regulate gating properties of the channel and resting membrane properties of DRG neurons [53]. The Nav 1.3 channels up-regulated following axotomy display rapid activation and inactivation [31,33]. It was suggested that the increased recovery rates from inactivation of Nav 1.3 channels expressed along the axon after axotomy compared to Nav 1.7 channels present under control conditions in small DRG neurons, could contribute to increased excitability of DRG neurons under neuropathic pain conditions [30]. Presynaptic Nav channels modulate the presynaptic action potential, subsequent Ca^2+ ^influx and thus transmitter release. Increased expression of TTX-S channels at the presynaptic ending could thus lead to amplification of the presynaptic potential and increased Ca^2+ ^influx and glutamate release [54]. Our results support the hypothesis that presynaptic TTX-S Nav channels on spinal cord primary afferent endings could mediate increased transmitter release and thus contribute to neuropathic pain hypersensitivity.

Our results with lidocaine application showed involvement of presynaptic TTX-R Nav channels in regulation of mEPSC frequency recorded in superficial dorsal horn neurons. There was no significant difference between the control and axotomized animals. Minimal effect of lidocaine application on the mEPSC frequency in neurons from axotomized slices after TNFα incubation suggested reduced participation of TTX-R Nav channels under these conditions. Downregulation of Nav 1.8 channel mRNA expression [32] and TTX-R sodium currents [33] has been shown in DRG neurons following sciatic nerve axotomy. In our preparation TTX-R Nav channel involvement in regulation of mEPSCs frequency was significantly reduced after the TNFα treatment, suggesting important regulatory role of this cytokine. In contrast, Jin and Gereau (2006) demonstrated enhancement of TTX-R sodium currents in cultured DRG neurons after acute TNFα application. This discrepancy is most likely due to different experimental conditions between the DRG cultures and the spinal cord slices with only central branch of the primary afferent present, different duration of the TNFα application and altered regulation of TTX-R Nav channels in injured and uninjured DRG neurons by TNFα [38,50].

In our experiments there was only a small reduction of the mean sEPSC amplitude after TTX application, irrespective of the TNFα treatment (control slices ~93%, axotomy ~86%). This would suggest that there was very low proportion of sEPSC present due to propagation of action potentials in the superficial DH neurons in our spinal slice preparation with cut dorsal roots before the TTX treatment, in contrast to preparations with intact neuronal circuits as in hippocampal slice preparation [55]. The TNFα treatment did not induce any significant change in sEPSC or mEPSC mean amplitude in the recorded neurons, similar to our previous results in control animals [16] and in agreement with previous finding [13,15,56]. However, enhancement of AMPA mediated postsynaptic currents by TNFα was shown in hippocampal neurons due to increased expression of surface AMPA receptor [57]. In the spinal cord, TNFα dependent AMPA receptor trafficking was demonstrated in association with peripheral inflammation [58] and cell death following spinal cord injury [59]. Potentiation of AMPA induced currents by TNFα was reported also in the spinal cord DH neurons in control slices [13], while two other studies did not find any TNFα modulation of AMPA induced currents in DH neurons [15,56].

Our previous experiments on superficial DH neurons done under the same experimental conditions showed that application of 10 μM OLDA solution was needed to increase mEPSC frequency due to specific TRPV1 receptor activation, while lower OLDA concentrations did not have an effect [39]. The OLDA concentration needed to activate presynaptic TRPV1 receptors decreased dramatically to 0.2 μM after PKC activation by phorbol esters and in a model of peripheral inflammation [39]. Results in this paper demonstrate increased sensitivity of spinal presynaptic TRPV1 receptors to endogenous agonist OLDA following sciatic nerve transection. We suggest that this responsiveness to low concentration (0.2 μM) OLDA solution could be mediated by phosphorylation or up-regulation of presynaptic TRPV1 receptors [60]. One of the mechanisms involved could be also increased expression of TNFR1 receptors in the DRG neurons following the nerve lesion [11]. In the experiments described in this paper, the increase of mEPSC frequency after the OLDA application was comparable in the neurons recorded after axotomy irrespective of the TNFα treatment. This is in contrast to our previous results in control slices, where TNFα treatment induced response to low concentration (0.2 μM) OLDA, not present in controls [16]. The lack of TNFα incubation effect in slices after axotomy may be due to already sensitized TRPV1 receptors present at the presynaptic endings. Decreased expression of TRPV1 receptors after axotomy could also play a role [61].

## Conclusions

Our results support an important regulatory role of the proinflammatory cytokine TNFα in nociceptive processing at the spinal cord DH following sciatic nerve section. We have demonstrated modulation of presynaptic TTX sensitive sodium channel activity and increased transmitter release by TNFα together with increased sensitivity of presynaptic TRPV1 receptors to endogenous agonist. These mechanisms could significantly affect synaptic transmission in the spinal cord DH after nerve injury and contribute to neuropathic pain development or maintenance.

## Competing interests

The authors declare that they have no competing interests.

## Authors' contributions

JP conceived and designed the study, DS and VN performed and analyzed the experiments. DS and JP drafted the manuscript. All authors have read and approved the final version of the manuscript.
